# Porcine lungs perfused with three different flows using the 8-h open-atrium cellular *ex vivo* lung perfusion technique

**DOI:** 10.3389/fbioe.2024.1357182

**Published:** 2024-06-25

**Authors:** Sana N. Buttar, Hasse Møller-Sørensen, Michael Perch, Hannelouise Kissow, Thomas N. B. Lilleør, Rene H. Petersen, Christian H. Møller

**Affiliations:** ^1^ Department of Cardiothoracic Surgery, Copenhagen University Hospital, Rigshospitalet, Copenhagen, Denmark; ^2^ Department of Clinical Medicine, University of Copenhagen, Copenhagen, Denmark; ^3^ Department of Cardiothoracic Anaesthesiology, Copenhagen University Hospital, Rigshospitalet, Copenhagen, Denmark; ^4^ Department of Cardiology, Copenhagen University Hospital, Rigshospitalet, Copenhagen, Denmark; ^5^ Department of Biomedical Sciences, University of Copenhagen, Copenhagen, Denmark

**Keywords:** *ex vivo* lung perfusion, cellular/acellular perfusate, open/closed atrium, prolonged *ex vivo* lung perfusion, *ex vivo* lung perfusion duration, *ex vivo* lung perfusion strategies

## Abstract

The number of lung transplantations is limited due to the shortage of donor lungs fulfilling the standard criteria. The *ex vivo* lung perfusion (EVLP) technique provides the ability of re-evaluating and potentially improving and treating marginal donor lungs. Accordingly, the technique has emerged as an essential tool to increase the much-needed donor lung pool. One of the major EVLP protocols, the Lund protocol, characterized by high pulmonary artery flow (100% of cardiac output [CO]), an open atrium, and a cellular perfusate, has demonstrated encouraging short-EVLP duration results. However, the potential of the longer EVLP duration of the protocol is yet to be investigated, a duration which is considered necessary to rescue more marginal donor lungs in future. This study aimed to achieve stable 8-h EVLP using an open-atrium cellular model with three different pulmonary artery flows in addition to determining the most optimal flow in terms of best lung performance, including lung electrolytes and least lung edema formation, perfusate and tissue inflammation, and histopathological changes, using the porcine model. EVLP was performed using a flow of either 40% (*n = 6*), 80% (*n = 6*), or 100% (*n = 6*) of CO. No flow rate demonstrated stable 8-h EVLP. Stable 2-h EVLP was observed in all three groups. Insignificant deterioration was observed in dynamic compliance, peak airway pressure, and oxygenation between the groups. Pulmonary vascular resistance increased significantly in the 40% group (p < .05). Electrolytes demonstrated an insignificant worsening trend with longer EVLP. Interleukin-8 (IL-8) in perfusate and tissue, wet-to-dry weight ratio, and histopathological changes after EVLP were insignificantly time dependent between the groups. This study demonstrated that stable 8-h EVLP was not feasible in an open-atrium cellular model regardless of the flow of 40%, 80%, or 100% of CO. No flow was superior in terms of lung performance, lung electrolytes changes, least lung edema formation, minimal IL-8 expression in perfusate and tissue, and histopathological changes.

## Introduction

Lung transplantation is an established treatment for non-malignant end-stage pulmonary disease (Aigner et al., 2012). However, the intervention remains limited due to the scarcity of donor lungs meeting the standard criteria for transplantation (Aigner et al., 2012). *Ex vivo* lung perfusion (EVLP) has emerged as a novel method to expand the donor pool by evaluating, improving, and treating marginal donor lungs that otherwise would have been rejected (Ali et al., 2023).

Currently, there are three different EVLP systems and protocols—Lund, Toronto, and Organ Care System—used in clinical settings (Steen et al., 2007; Cypel et al., 2008; Loor et al., 2017; Sommer et al., 2019). Of these, two of the major protocols, Lund and Toronto, are applied in majority of the institutions (Steen et al., 2007; Cypel et al., 2008). The Toronto protocol, characterized by low pulmonary artery flow (40% of cardiac output [CO]), a closed atrium, and an acellular perfusate (LCAA), is the most comprehensively studied and continuously evolving protocol with promising results after short (4 h in human lungs) and long durations of EVLP (12–24 h to 3 days in pig lungs) (Cypel et al., 2008; Yeung et al., 2017; Takahashi et al., 2021; Ali et al., 2022). In contrast, the Lund protocol, distinguished by high pulmonary artery flow (100% of CO), an open atrium, and a cellular perfusate (HOAC), has demonstrated encouraging results after a short period of EVLP (1–4 h in human and pig lungs); however, the potential of an effective longer EVLP duration needs to be further investigated (Erasmus et al., 2006; Niikawa et al., 2020). There is no consensus as to which strategy is the most beneficial in terms of post-EVLP lung performance, least edema formation, perfusate and tissue inflammation, histopathological changes, and electrolyte balance in the perfusate, which has been associated with lung function deterioration, particularly pertaining to prolonged EVLP (Koike et al., 2011; Lin et al., 2018; Nilsson et al., 2018). However, one study has demonstrated no difference between the strategies, although with slightly more pronounced lung edema in the LCAA model in a pig model (Nilsson et al., 2018). One pivotal difference, which has been persistently debated between these two strategies, is the pulmonary artery flow at which lungs are perfused with no clarification of the most optimal EVLP perfusate flow (Nilsson et al., 2018; Beller et al., 2020). Nevertheless, there is a growing recognition that the future EVLP system, including perfusate flow, needs to facilitate prolonged lung preservation in order to utilize advanced diagnostics to predict transplant outcome and perform therapeutic interventions within the platform so that more marginal lungs can be rescued (Ali et al., 2023). While the LCAA model continues to successfully attain some of these objectives (Cypel et al., 2008; Yeung et al., 2017; Takahashi et al., 2021; Ali et al., 2022), the ability of the HOAC protocol has yet to demonstrate advancement toward prolonged lung preservation (Erasmus et al., 2006; Niikawa et al., 2020).

The purpose of this experimental study was to use the Vivoline LS1 system with the open-atrium cellular EVLP strategy in order to 1) determine the potential of 8-h EVLP and 2) examine solely the effect of three different perfusate flows (40%, 80%, and 100% of estimated CO) on the lung performance, including lung electrolytes, lung edema formation, perfusate and tissue inflammation, and histopathological changes, using the porcine model.

## Materials and methods

### Animals

Domestic female pigs (50–60 kg) were used under an experimental protocol approved by the Institutional Animal Care and Use Committee (IACUC) at the University of Copenhagen, 2021-15-0201-01045. The pigs were housed in an animal facility affiliated with the University of Copenhagen, where they received care in compliance with the “Principles of Laboratory Animal Care,” formulated by “Danish Animal Research Legislation.”

### Lung harvest, preservation, and preparation for EVLP

All pigs were pre-medicated with Zoletil (Zoletil^®^ Vet, Virbac, Denmark) (0.14 mL/kg). General anesthesia was achieved with 5 mg/kg 2,5% sodium pentobarbital (Pentothal, MTC Pharmaceuticals, Cambridge, Canada) intravenously (i.v.). Anesthesia was maintained with 15 mg/kg/h i.v. propofol (Propolipid^®^ 10 mg/mL, Fresenius Kabi AB, Uppsala, Sweden) and 15 μg/kg/min i.v fentanyl (50 μg/mL, Hameln Pharma plus GMBH, Hameln, Saksa/Germany).

The pigs were intubated with an endotracheal tube of size 8.0 mm (Portex, Sims, Markham, Canada) through the mouth. Ventilation was performed using a volume-controlled ventilator (Servo Ventilator, Siemens-Elema Ab, Sweden). The fraction of inspired oxygen (FiO_2_) was 50%. The respiratory rate was adjusted to an end-tidal CO_2_ of 5–5.5 kPa. The tidal volume was set at 6–8 mL/kg, and positive end-expiratory pressure (PEEP) was set at 5 cm H_2_O. The baseline arterial blood gas measurements were obtained before sternotomy.

After sternotomy, main pulmonary artery (PA) cannulation, and washed red blood cell collection, the aorta was clamped upon arrhythmia. The PA was antegradely perfused with 2 L of cold Perfadex (Perfadex, XVIVO Perfusion AB, Gothenburg, Sweden). At this stage, the pigs were pronounced dead (cardiac arrest with no cardioplegia and no volume/hemodynamic and ventilation resuscitation). The lungs were harvested, weighted, stored in cold saline, and kept on ice. After 2 h of cold ischemia, the lungs were cannulated and connected to the EVLP circuit. Washed red blood cells were prepared in a cell saver after exsanguination. The pigs were randomly assigned each day to an EVLP target flow of either 40% of CO (used in the Toronto protocol) (*n* = 6), 80% of CO (chosen arbitrary) (*n* = 6), or 100% of CO (used in the Lund protocol) (*n* = 6) (section details are given in Supplementary Material S1A).

### EVLP procedure

**FIGURE 1:**
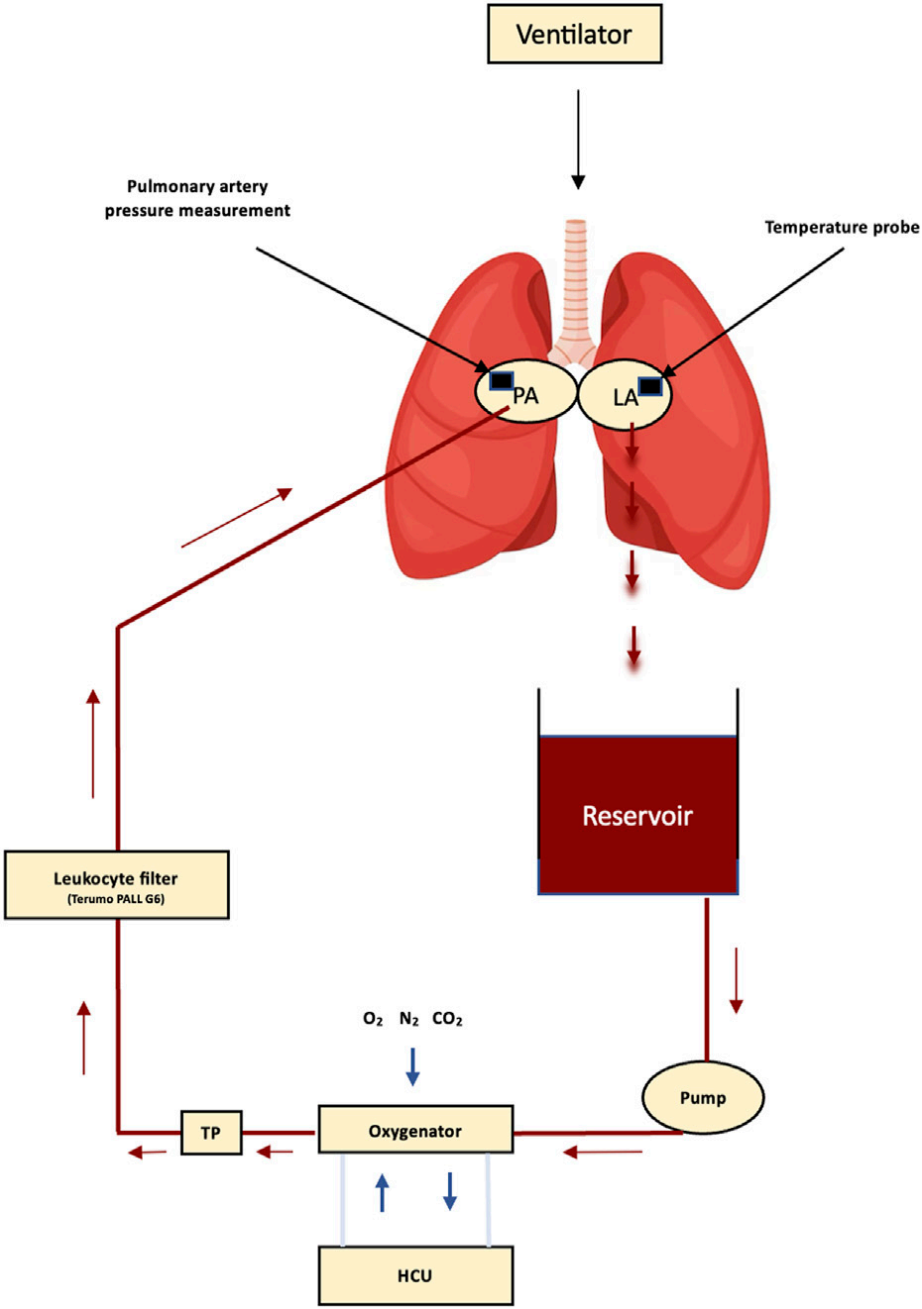
Schematic diaphragm of the components in a Vivoline LS1 circuit (Vivoline Medical AB, Lund, Sweden). PA, pulmonary artery; LA, left atrium; HCU, heater–cooler unit; TP, temperature probe.

Vivoline LS1 (Vivoline Medical AB, Lund, Sweden) was used for this experiment (Figure 1). The system was primed with 2 L of STEEN solution (XVIVO Perfusion AB, Gothenburg, Sweden), heparin, meropenem, methylprednisolone, and washed red blood cells to a hematocrit level of 10%–15% (Supplementary Material S1B). Rewarming of the lungs with a target temperature of 37°C was commenced after de-airing of the PA cannula and the subsequent connection of the lungs to the circuit. The antegrade flow was initiated at 10% of estimated CO (pigs: CO = 70 mL/min/kg (Wallinder et al., 2014)), with a gradual increase to either 40% , 80%, or 100% of estimated CO for the reconditioning phase at FiO_2_ 0.5 at 37°C (Table 1). Lung evaluation was performed at FiO_2_ 1.0 every 2 h, while evaluation with FiO_2_ 0.21 was conducted in the last hour before EVLP termination. Ventilation was started at 32°C, with incremental changes in the ventilatory parameters according to the temperature and EVLP phases (Table 1). The baseline lung parameters (dynamic compliance [DC], pulmonary vascular resistance [PVR], peak airway pressure [PawP], and oxygenation) and perfusate gasses (pH, sodium, potassium, lactate, and glucose) were taken after 10 min of target flow (hour 0), with subsequent hourly reconditioning assessments (Table 1). The lung parameters and perfusate gasses were evaluated every 2 h (Table 1). If needed, perfusate gasses were corrected on an hourly basis as follows: pH was maintained between 7.35 and 7.45 with 1 mL of isotonic trometamol (Addex-THAM 20 mL, Fresenius Kabi AB, Uppsala, Sweden) for every unit below zero in the base excess (Wallinder et al., 2014). Calcium was maintained at >3 mmol/L with 10 mL calcium gluconate. Glucose was maintained at >5 mmol/L with 100 mg/mL glucose (Fresenius Kabi). All the lung function parameters were recorded from the EVLP and respiratory monitors.

**TABLE 1:** Strategy of EVLP and ventilation.

EVLP phases (minutes)	Rewarming (30–45)		Reconditioning (60)	Evaluation (10)
Temperature (°C)	25	32	37	37
Flow (% of 70 mL/min/kg)	10 (shunt closed)	25	Target flow	100
Ventilation	None	Initiated with	Adjusted to	Re-adjusted to
		FiO_2_: 50	FiO_2_: 50	FiO_2_: 100 and 21
		PEEP: 5	PEEP: 5	PEEP: 5
		PawP: <20	PawP: <25	PawP: <25
		TV: 2–4	TV: 4–6	TV: 6–8
		RR: 12	RR: 12	RR: 12
Oxygen (O_2_ 21%)	None	Connected	Connected	Disconnected
Gas	None	74% N, 5% CO_2_		93% N, 7% CO_2_

EVLP, *ex vivo* lung perfusion; FiO_2_, fraction of inspired oxygen (%); PEEP, positive end-expiratory pressure (cm H_2_O); PawP, peak airway pressure (cm H_2_O); TV, tidal volume (mL/kg); RR, respiratory rate (per minute); O_2,_ oxygen; N, nitrogen; CO_2_, carbon dioxide.

A successful prolonged EVLP was pre-defined as 8 h if both of the following criteria were met: 1) DC > 15 mL/cm H_2_O, which was also considered a parameter of stable EVLP, and 2) an achievable target perfusion flow. If none of these two parameters were accomplished during the reconditioning hour, a decision was made to terminate the EVLP toward the end of that hour before the evaluation phase. EVLP was continued if one of these parameters was feasible until the 8-h mark in order to observe the trends.

### Perfusate and lung sample collection

Perfusate samples for cytokine analysis were collected every hour from the left atrium port and stored at −80°C until analysis. Both the lung sides and all lobes were used to collect the lung tissue for cytokine analysis and microscopic evaluation. Once a lung side and a lobe were chosen arbitrarily for each case, the lung tissues were collected from the same lung side and lobe before and after the EVLP. One portion of the collected lung tissue (before and after) was snap-frozen on dry ice and stored at −80°C for cytokine analysis. A separate portion of the collected lung tissue (before and after) was fixated in formalin for microscopic evaluation.

### Histology of the lung samples

The lung samples were fixed in 10% buffered formalin for 24 h, embedded in paraffin, sectioned into a thickness of 5 µm, and stained by hematoxylin and eosin before the evaluation of pathological changes under light microscopy. Histopathologic grading of lung injury was assessed using the parameters of interstitial edema, intra-alveolar edema, arteriolar thickening, vascular thrombosis, hemorrhage, and cell infiltration (Medeiros et al., 2012). The severity of these findings was graded on a 4-point scale as follows: 0, absent; 1, mild; 2, moderate; and 3, severe (Medeiros et al., 2012). The tissue slices were blindly evaluated by two independent reviewers (HK and SNB). Any discrepancies were resolved through consensus.

### Wet-to-dry weight ratio of the lung samples

Immediately after EVLP termination, a 1 × 1 × 1-cm biopsy was taken from the right or left lower lobe. The ratio of the weights of the portions before and after drying (60°C in an oven for 24 h) was calculated to evaluate lung edema after EVLP (Wallinder et al., 2014).

### Cytokine analysis in the perfusate and lung samples

Inflammatory markers in the perfusate and lung tissue were measured using commercially available enzyme-linked immunosorbent assay (ELISA) kits for porcine cytokine interleukin (IL)-8, IL-10, tumor necrosis factor-⍺ (TNF-⍺), and hypoxia inducible factor-1⍺ (HIF-1⍺). The kits were used according to the manufacturer’s instructions (section details are given in Supplementary Material S1C).

### Statistical analysis

Continuous data are presented as the mean ± standard deviation or standard error of the mean. Analysis of variance (ANOVA) was performed to compare the differences among more than two groups. When the statistical significance was indicated by ANOVA, a pair-wise *post hoc* test (Tukey’s test) was performed to identify the differences between the groups. Changes within the groups were assessed by paired *t*-tests. Perfusion hours were analyzed using Kaplan–Meier and log-rank tests. Statistical analysis was derived from the time of the start of EVLP (hour 0) till the EVLP termination of each lung. To address the truncated data variation, each outcome was divided by its EVLP hour, followed by the average analysis of each group. A *p*-value <0.05 was considered statistically significant. All statistical analyses were performed in RStudio (version 2022.07.2).

## Results

**FIGURE 2:**
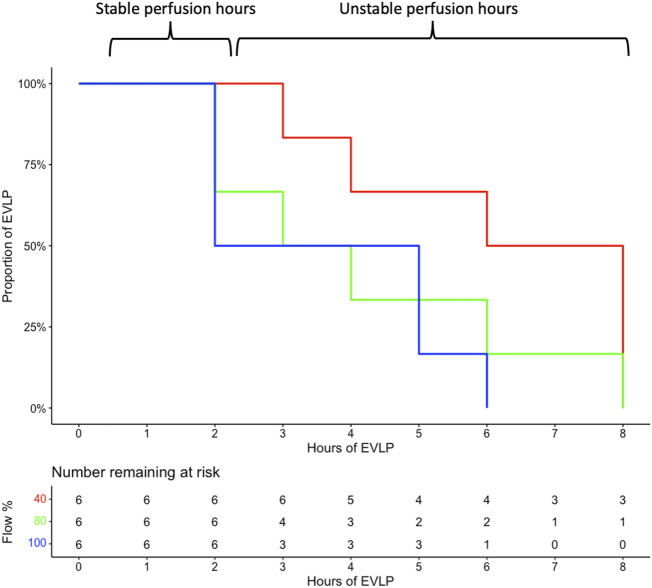
Perfusion hours were 8, 8, 4, 8, 6, and 3 h in the 40% group; 4, 3, 2, 2, 6, and 8 h in the 80% cohort; and 2, 5, 6, 2, 2, and 5 h in the 100% group (recorded as 8-h time points in all groups). EVLP, *ex vivo* lung perfusion; %, percent.

**FIGURE 3:**
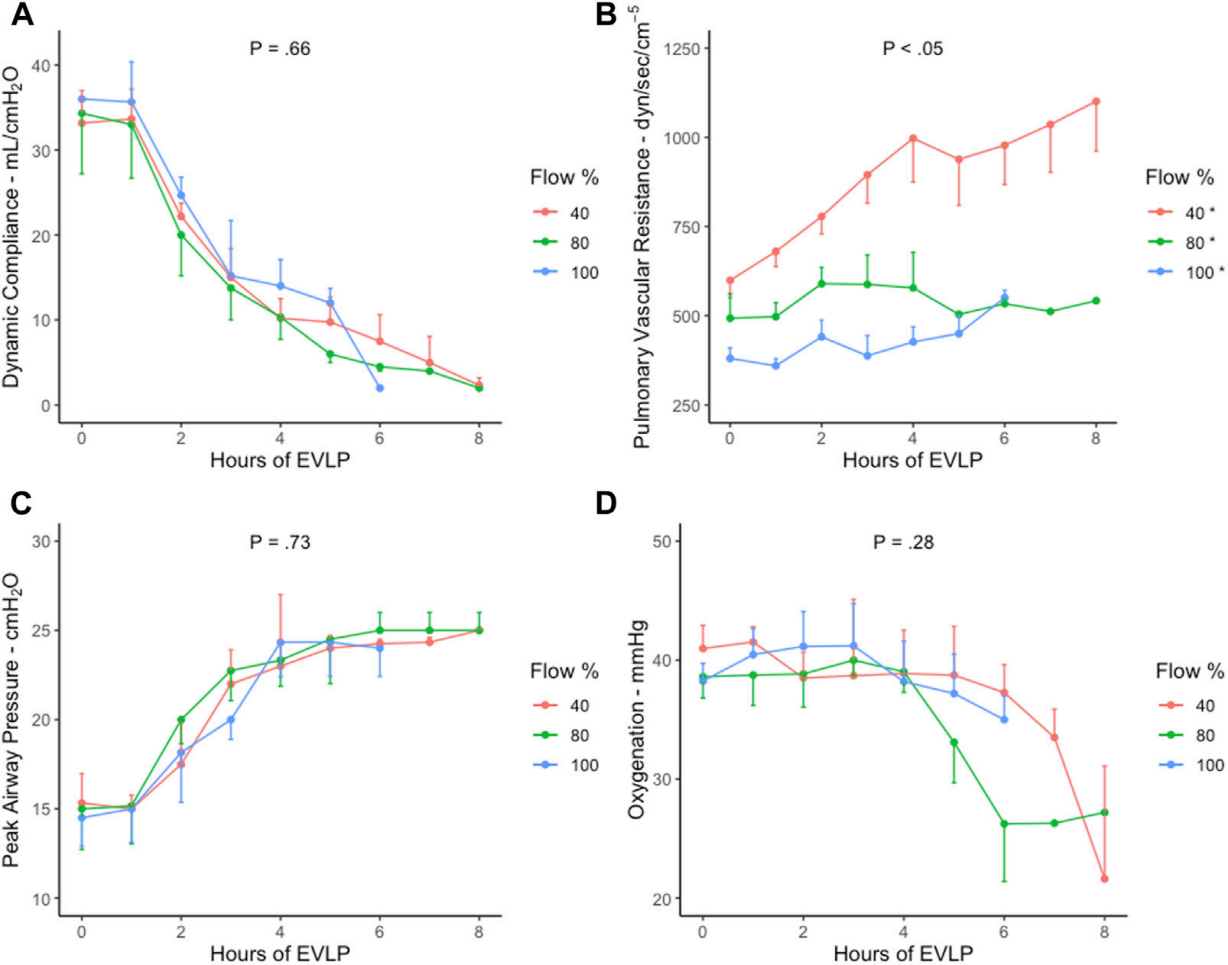
Lung parameters during EVLP perfusion in 40% (*n* = 6), 80% (*n* = 6), and 100% (*n* = 6) flow rate groups with standard error of the mean. **(A)** Dynamic compliance, **(B)** pulmonary vascular resistance, **(C)**, peak airway pressure, and **(D)** oxygenation. *, Significance between the groups using Tukey’s test (40% vs. 80% and 100%: *p* < .05; 80% vs. 100%: *p* = 0.02). *p*, *p*-value; EVLP, *ex vivo* lung perfusion; %, percent.

No significant difference in pig body weight (40%: 54.7 ± 4.5 kg; 80%: 51.2 ± 1.9 kg; and 100%: 54.5 ± 2.1 kg; *p* = 0.9) and arterial PO_2_/FiO_2_ at lung retrieval (40%: 592.0 ± 40.6 mmHg; 80%: 608.3 ± 40.5 mmHg; and 100%: 522.0 ± 82.6 mmHg; *p* = 0.06)) was found between the groups. Perfusion hours based solely on the ability to run the target perfusate flow were 6.17 ± 2.23 in 40%, 4.17 ± 2.4 in 80%, and 3.67 ± 1.9 in the 100% group (*p* = 0.10) (Figure 2). Stable perfusion hours were only observed in the first 2 h in all groups (Figures 2, 3).

The lung parameters were stable in the first 2 h, followed by a worsening trend throughout the EVLP period in all the groups (Figures 3A–D). No group achieved stable 8-h EVLP as DC was observed to insignificantly decrease <15 mL/cm H_2_O after 2 h in all the groups (*p* = 0.66) (Figure 3A). PVR increased significantly between all the groups, with the highest increase in the 40% cohort (Tukey’s test: 40% vs. 80% and 100% groups, *p* < 0.05) (Figure 3B). A similar increasing trend was demonstrated by PawP in all the groups (*p* = 0.73) (Figure 3C). Oxygenation was insignificantly stable for the first 4 hours, with a subsequent decrease in all the groups (*p = .28*) (Figure 3D). All lung parameters were insignificantly exacerbated in all the groups with a FiO_2_ increase (50%–100%) and decrease (50%–21%).

**FIGURE 4:**
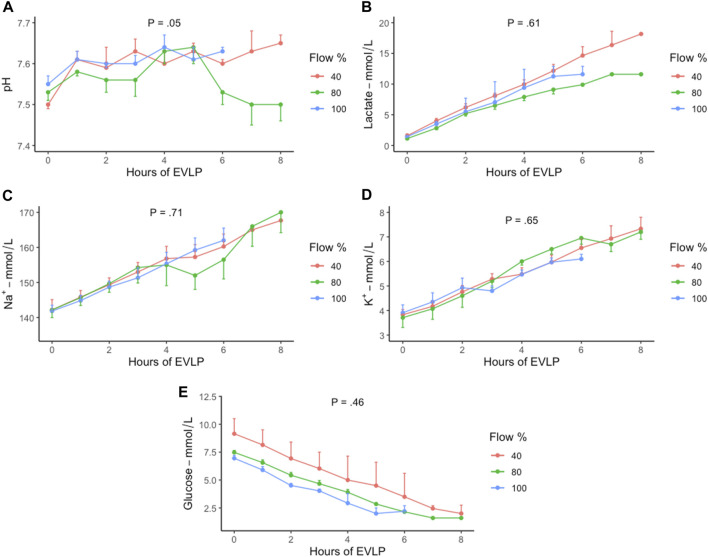
Trend of electrolyte concentration during EVLP perfusion in 40% (*n* = 6), 80% (*n* = 6), and 100% (*n* = 6) flow groups with standard error of the mean. **(A)** pH, **(B)** lactate, **(C)** sodium, **(D)** potassium, and **(E)** glucose. EVLP, *ex vivo* lung perfusion; %, percent.

Corresponding electrolyte concentrations were also noted to aggravate in all the groups (Figures 4A–E). The pH, lactate, sodium, and potassium values increased insignificantly between the groups (*p* = 0.05, *p* = 0.61, *p* = 0.71, and *p* = 0.65, respectively [Figures 4A–D]), whereas the glucose values decreased insignificantly in all the groups (*p* = 0.46) (Figure 4E).

**FIGURE 5:**
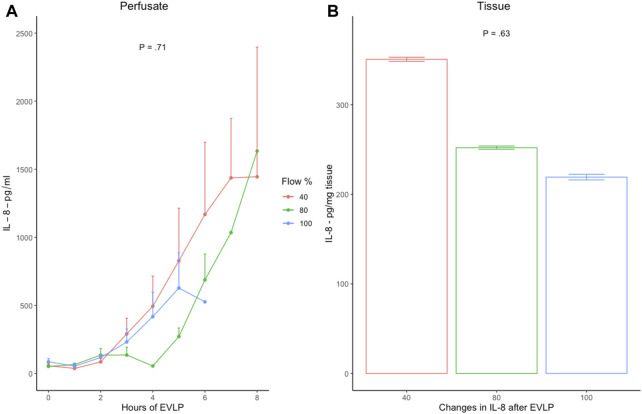
**(A)** Hourly pro-inflammatory IL-8 levels in the perfusate with standard error of the mean, and **(B)** mean changes in IL-8 production in lung tissue from baseline (H_0_) and end of EVLP (H_T_) in 40% (*n* = 6), 80% (*n* = 6), and 100% (*n* = 6) flow groups. Error bars represent standard error values. IL-8, interleukin-8; EVLP, *ex vivo* lung perfusion; *p*, *p*-value.

Pro-inflammatory IL-8 levels in the perfusate and tissue demonstrated a time-dependent increase, with no difference between or within the groups (Figures 5A, B). Due to the repeated gross difference in ELISA duplicate testing of IL-10, TNF, and HIF concentration levels, no reliable statistical analysis could be performed for these cytokines.

**FIGURE 6:**
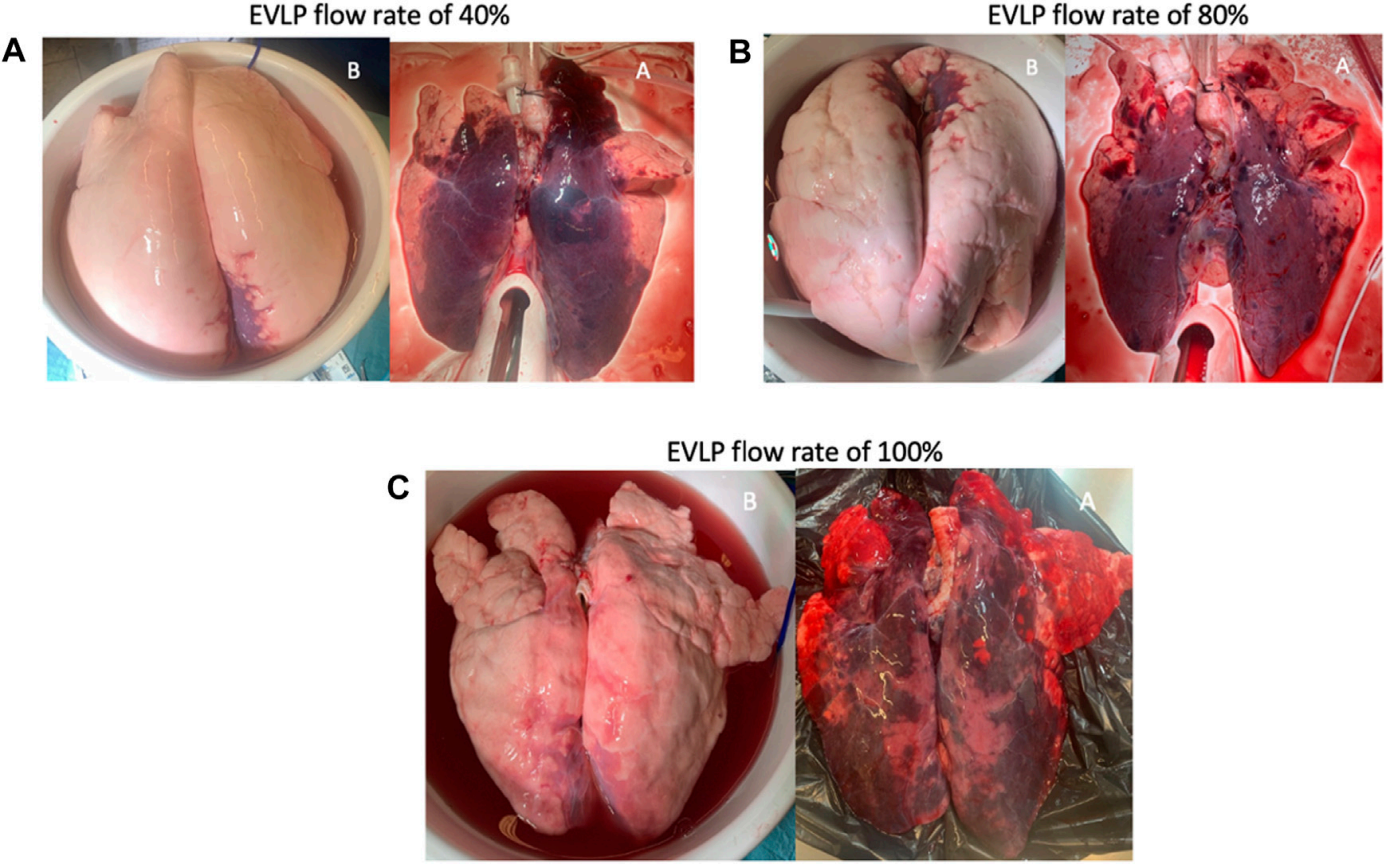
Appearance of lungs before **(B)** and after **(A)** 6-h EVLP in the **(A)** 40% flow rate group, **(B)** 80% flow rate group, and **(C)** 100% flow rate group. All lungs were perfused in a supine position. EVLP, *ex vivo* lung perfusion.

**FIGURE 7:**
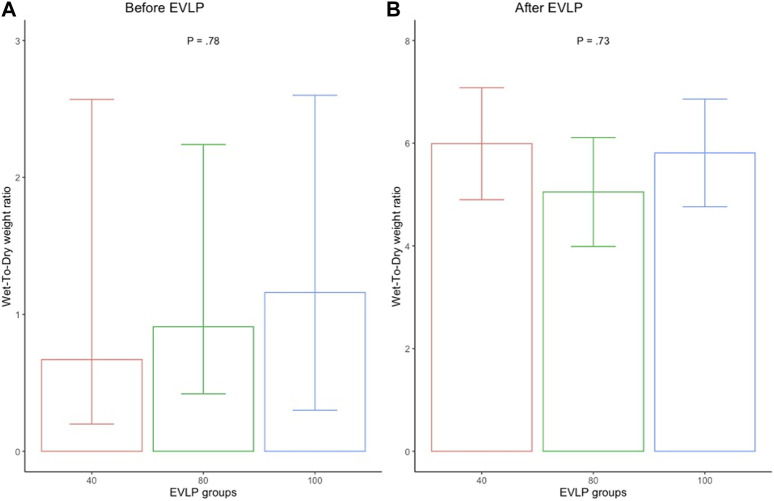
Mean wet-to-dry weight ratio of the perfused lungs in 40% (*n* = 6), 80% (*n* = 6), and 100% (*n* = 6) flow groups before **(A)** and after **(B)** the EVLP. Error bars represent standard error values. *p*, *p*-value; %, percent.

**FIGURE 8:**
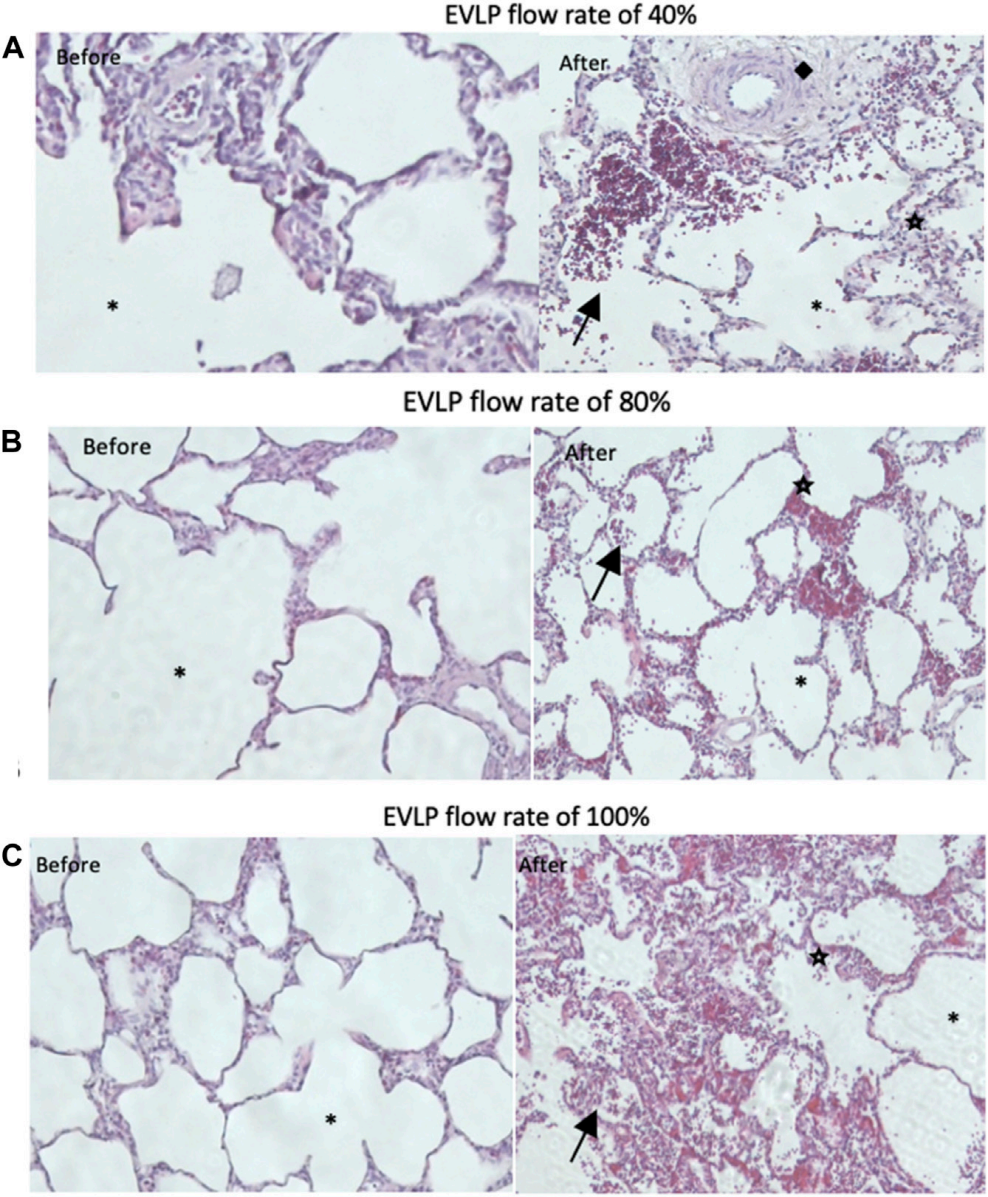
General comparison of histopathological lung injury before and after EVLP between the **(A)** 40% flow rate group, **(B)** 80% flow rate group, and **(C)** 100% flow rate group. ◆, arteriolar thickening, 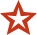
cell infiltration, (arrow) hemorrhage, and *, alveolar. EVLP, *ex vivo* lung perfusion.

Edema formation was visibly attenuated in all the groups after EVLP (Figures 6A–C). The wet-to-dry weight ratio (WDR) increased in all three groups after EVLP, although it was time-dependently slightly higher in the 40% group (*p =* 0.73) (Figures 7A, B). Time-dependent cumulative pathological scoring of lung injury demonstrated no significant difference between or within the groups before and after EVLP (Figures 8A–C).

Notably, results from 3-h EVLP and onward are cautiously interpreted on account of the conspicuous variation in the number of pigs between the groups.

## Discussion

The mounting body of evidence demonstrates reproducible stable prolonged EVLP with the LCAA strategy (Cypel et al., 2008; Yeung et al., 2017; Takahashi et al., 2021; Ali et al., 2022). This is in contrast to the HOAC approach, where the potential of stable prolonged EVLP remains undetermined with limited robust and consistent data (Erasmus et al., 2006; Niikawa et al., 2020). The longest assessed HOAC EVLP duration is 6 h in porcine lungs, with results indicating high PVR and maximum ventilation pressure, suggestive of lung injury (Erasmus et al., 2006). However, unpublished data of HOAC EVLP have been cited to perfuse porcine lungs for 8 h without lung edema formation, but conclusive evidence is yet to be reported (Wierup et al., 2006). In the present study, stable 8-h perfusion was not feasible with either lower perfusate flow (LPF) (40%) or higher perfusate flow (HPF) (80% and 100%). All three perfusate flows demonstrated deteriorating trends in DC, PVR, PawP, and oxygenation, which is suggestive of severe lung injury. Several deleterious factors may have contributed to damage the integrity of the lung structure. In the HPF groups, the most plausible explanation may be the exposure of excessive hydrostatic pressure, which encouraged mechanistic damage of pulmonary vasculature, resulting in greater fluid permeability, thus leading to advanced lung edema formation (Okamoto et al., 2019; Beller et al., 2020). This mechanism has led to the adoption of LPF in the LCAA strategy (Cypel et al., 2008). Whether LPF alone has beneficial effects on prolonging LCAA EVLP success is not investigated; however, studies have demonstrated that even lower perfusate flow (20% of CO) in the LCAA system may further reduce lung edema (Beller et al., 2020). In the present study, reducing the perfusate flow was equally inferior as HPFs in terms of DC, PawP, and oxygenation. Interestingly, PVR as an isolated parameter became significantly higher in the LPF group than in the HPF groups. These findings may be because the lower flow in combination with the open atrium creates a cyclical open–close phenomenon at the capillary level with ventilation exacerbated at low after-load pressures, leading to altered permeability and edema formation (Broccard et al., 2002; Peták et al., 2002; Linacre et al., 2016). Another potential explanation may be that the absence of venous after-load pressure in the pulmonary circulation altered the distribution in the lung perfusion zones with either the lack or overflow of the perfusion, thus contributing to the damage of the lung (Peták et al., 2002; Linacre et al., 2016). Interestingly, our LPF results are in contrast with those of limited previous studies demonstrating stable lung parameters for more than 8 h in a low-flow, open-atrium, cellular (LOAC) strategy (50% of CO) (Loor et al., 2017; Sommer et al., 2019). This may be because these studies used an EVLP system (Organ Care System), which is notably different in terms of the circulatory, ventilatory, and priming setups from a Vivoline LS1 system. In addition, their reconditioning protocols (replenishment regime and recruitment maneuverers) are significantly diverse, which may explain their favorable outcomes compared to our study. Nevertheless, our LPF findings are supported by another study that demonstrated an increase in PVR and peak inspiratory pressure and a decrease in compliance and oxygenation in a setting of LPF with an open atrium compared to a closed atrium (Linacre et al., 2016). However, an increase in PVR alone in our LPF group cannot be fully delineated, especially given that the LPF group was the only group in which most lungs reached unstable 8-h EVLP. One may explain this contradiction with insufficient generation of PVR in these specific lungs to obstruct the target flow. However, the exact mechanism behind it cannot be thoroughly elucidated except the known fact that PVR determination is based on the perfusate flow rate, which may have influenced the higher PVR in the lower perfusate flows.

Preserved lung viability after short EVLP durations has been demonstrated in LCAA and HOAC strategies with promising results after transplantation (Wierup et al., 2006; Cypel et al., 2011; Henriksen et al., 2014; Nilsson et al., 2019; Buttar et al., 2024). In our study, lung parameters were within acceptable values in the first 2 h, suggesting a stable 2-h EVLP. However, deterioration was already apparent during the second hour in all perfusate flows, indicating edema formation with a continuing decrease in lung performance in the subsequent hours. These findings are in line with the fact that the HOAC model was proposed as a method to evaluate marginal lungs within a short duration (1–2 h) of EVLP due to the concern of lung damage associated with concomitant high hydrostatic pressure (Steen et al., 2007; Okamoto et al., 2019; Beller et al., 2020). Nevertheless, 4-h stable EVLP with the HOAC model in pig lungs and 4-h stable EVLP with the LPF open-atrium cellular model in human lungs have been demonstrated (Valenza et al., 2014; Nilsson et al., 2018). Interestingly, in the present study, oxygenation as an isolated parameter was found to be stable for 4 h. This may indicate that oxygenation alone may not be a reliable indicator of lung viability. It has been demonstrated that lung assessment conducted solely based on oxygenation can be misleading as its deterioration can be delayed in an *ex vivo* setting (Yeung et al., 2012). Therefore, an evaluation of other physiologic parameters such as DC, PawP, and PVR is crucial to obtain a full picture of the functional capacity of the lungs (Cypel et al., 2009; Yeung et al., 2012; Spratt et al., 2017).

As all perfusate flows have performed equally unsatisfactorily in the present study, one may hypothesize on the lack of deleterious underlying factors. One of them could have been that cold ischemia time (CIT) of 2 h in a prolonged EVLP set point was not sufficient in terms of induced lung injury. To date, no pig study has compared different perfusate flows with 2-h CIT using the open-atrium cellular strategy in a prolonged time-point EVLP. However, studies with 2-h CIT, LPF, and 4-h cellular EVLP have demonstrated no difference in the development of lung edema compared with acellular perfusate (Roman et al., 2015). The same results have been demonstrated by studies with 24-h CIT, HPF, and 12-h cellular EVLP compared with acellular perfusate (Becker et al., 2016; Steinmeyer et al., 2018). This indicates that CIT duration may not influence the true difference if all cases have been subjected to the same CIT duration regardless of EVLP duration, perfusate flow, or perfusate composition. Thus, our results cannot be explained by a short 2-h CIT as it is sufficiently harmful injury to the lungs. Nevertheless, longer CIT hours have been associated with diminishing reconditioning potential due to severe ischemia reperfusion injury (IRI) (Steinmeyer et al., 2018; Okamoto et al., 2019).

Another general key difference between LCAA and HOAC strategies is that perfusate in the LCAA model is periodically replenished, whereas the HOAC setup corrects perfusate composition in an hourly manner (Steen et al., 2007; Cypel et al., 2008). However, there is one study with the LOAC strategy that has also implemented a replenishment regime with promising results (Loor et al., 2017). In the present study, electrolytes demonstrated a steady deterioration in an hourly manner in short and long EVLP duration in all groups. These results may be because perfusate correction during the EVLP was not sufficiently attained despite hourly correction with an acceptable follow-up gas analysis. This suggests that perfusate components are consumed rapidly with longer EVLP; thus, the need for perfusate correction should be assessed multiple times within an hour and not be limited by one acceptable hourly correction, as demonstrated in a previous study (Sommer et al., 2019). Nevertheless, deterioration of electrolytes in prolonged EVLP is inevitable even with standard replenishment of the perfusate in the LCAA model, although the exacerbation is observed after 12 h (Cypel et al., 2008). Considering this factor, periodical replenishment of the perfusate appears to play one of the key roles in enabling stable prolonged EVLP rather than correcting electrolytes (Cypel et al., 2008; Loor et al., 2017). A recent study from the LCAA model group focuses on further stabilization of homeostasis with a demonstration of even longer stable EVLP, thus emphasizing the significance of sufficient perfusate constituents (Takahashi et al., 2021).

In lung IRI, immune cells are induced to generate inflammatory cytokines, which increase capillary permeability, resulting in lung edema (de Perrot et al., 2003; Den et al., 2010). In this study, pro-inflammatory cytokine (IL-8) in perfusate and tissue demonstrated a time-dependent increase in all groups with no difference between the groups. The increase in the IL-8 levels may correspond to a severe increase in capillary permeability and, thus, lung edema, as observed in all the groups. Previous studies with the HOAC model have demonstrated similar results; however, there is no prior evidence of the IL-8 trend in the low-flow cellular model (Nilsson et al., 2018; Okamoto et al., 2019). Nevertheless, IL-8 in the low-flow acellular setup demonstrates results similar to that of our low-flow cellular group (Nilsson et al., 2018), which is in line with severe edema formation supported by time-dependent EVLP results of WDR and post-EVLP histology between the groups.

The main limitation of this study is the discrepancy in EVLP hours between the groups, in particular, the difference in the number of remaining lungs on EVLP after 2 h. This inconsistency results in an inequitable comparison of the groups after 2 h. Given that one of the main purposes of the study was to demonstrate the potential of 8-h EVLP with the open-atrium cellular technique, we found the necessity to analyze data based on the cumulative EVLP hours of each group. Nonetheless, 2-h trends between the groups have been noted. Another limitation of this study is the small number of cases in addition to the lack of blinded randomization. Thus, our study does not provide definitive evidence of the infeasibility of 8-h EVLP and no difference in the development of lung edema between the three compared perfusate flows using the open-atrium cellular EVLP strategy. Lastly, the lungs were only flushed antegradely with 2 L of Perfadex, which may have had an adverse effect on the outcomes. Nevertheless, considering that all three flow groups were subjected to the same amount of Perfadex, one may have equalized the outcome effect across the groups.

In conclusion, 8-h EVLP is not feasible in an open-atrium cellular pig model in the presented EVLP setup regardless of perfusate flow of 40%, 80%, or 100% of CO. Nevertheless, this study demonstrates a stable 2-h EVLP within all three perfusate groups. No perfusate flow is superior in terms of lung performance, lung electrolyte changes, minimal IL-8 expression in perfusate and tissue, detrimental histopathological changes, and lung edema formation. Modifications in the applied EVLP protocol, in particular, the addition of a replenishment regime, may improve the ability of longer EVLP duration with better post-EVLP outcomes. Studies with equivalent long EVLP hours in each flow group are required to determine the true difference in the measured parameters between the perfusate flows.

## Data Availability

The raw datasets used and/or analysed during the current study are available from the corresponding author on reasonable request.

## Abbreviations

HOAC, High pulmonary artery flow, an Open Atrium, and a Cellular perfusate; HPF, high perfusate flow; LCAA, Low pulmonary artery flow, a Closed Atrium, and a Acellular perfusate; LOAC, Low pulmonary artery flow, an Open Atrium, and a Cellular perfuse; LPF, Low Perfusate Flow.
