# Supplementary material for: Porcine lungs perfused with three different flows using the 8-h open-atrium cellular ex vivo lung perfusion technique
Source: Front Bioeng Biotechnol. 2024 Jun 25;12:1357182. doi: 10.3389/fbioe.2024.1357182 (PMC11231398; doi:10.3389/fbioe.2024.1357182)
Supplement: Supplementary file 1 [file DataSheet1.docx]

**Supplementary Material:**

**A: Lung Harvest, Preservation, and preparation for EVLP**

Harvest and Preservation: After sternotomy, thymus removal and pericardium opening, the superior and inferior vena cava were encircled with silk ties. A bolus of 15.000 IE Heparin (LEO Pharmaceutical, Copenhagen, Denmark) was injected i.v. A 21 French cannula was inserted into the main pulmonary artery (PA) and secured with a 4-0 prolene (Ethicon, Peterborough, Canada) purse-string suture. Before clamping the aorta, autologous whole blood was drained from the superior vena cava using 32 F venous cannula into a reservoir bag (TransMedic Inc.) for priming the EVLP. Upon arrythmia, aorta was clamped, the superior and inferior vena cavae were ligated and the heart was arrested by initiation of in situ antegraded flush with 2 litres of cold low-potassium dextran glucose preservation (LPDG) solution (Perfadex, XVIVO Perfusion AB, Gothenburg, Sweden). Left atrial appendage was transected to relieve the heart and the lungs. Ventilation was continued throughout the extraction of the heart-lung block. The trachea was clamped with the lungs inflated with a sustained airway pressure of 15 cm H_2_O. After removal of the heart-lung block, the lungs were weighted and stored in cold saline at 4-8°C for 2 hours. The collected autologous blood was added 10.000 U Heparin (Hepalean, Leo Pharma Inc. Ajax, Canada) and washed in a cell saver (Haemonetics model no. 2005) to collect washed red cells.

Preparation: The heart was excised from the heart-lung block. The remnant of the left atrium was trimmed to be wide open to prevent pulmonary vein outflow obstruction and to keep left atrium pressure near 0 mmHg. A cannula was sewn to the pulmonary artery (PA) with a 4-0 mono-filament suture. A sized-matched silicon tube was secured to trachea with silk ties. After the lungs were connected to EVLP system, a temperature probe was sutured inside the left atrium. All ex vivo lung perfusion was performed in antegrade fashion.

**B: EVLP procedure**

Priming: Apart from 2 litres of STEEN solution (XVIVO Perfusion AB), Heparin 15.000 IE (LEO Pharmaceutical, Copenhagen, Denmark), Meropenem 100mg, Methylprednisolone 1g and salvaged blood cells 500-800ml (to a haematocrit of > 10%) were used for priming.

All lung parameters were recorded from the EVLP- and respiratory monitors.

**C: Cytokine analysis in Perfusate and Lung tissue**

Perfusates were stored in -80 ℃ in hourly manner for all 18 pigs and ready for cytokine analysis. Lung tissues (before - and after EVLP) were weighted separately for all 18 pigs before suspension of 0.5 ml of phosphate-buffered saline (PBS) in each tissue sample (weighing approximately 80-90 g each) followed by homogenization. The samples were then centrifuged at 5000 rpm for 10 minutes. The supernatants were collected and stored at -80 ℃ until cytokine analysis. Cytokine analysis for perfusate and tissue samples was performed using porcine cytokine enzyme-linked immunosorbent assay (ELISA) kits. The ELISA kits specific for porcine IL-8, IL-10, TNF-⍺ and HIF-1⍺ were purchased from Nordic BioSite. The lowest detection limits of kits were as follows: 125 picogram (pg)/millilitre (ml) (IL-8), 6.25 pg/ml (IL-10), 31.25 pg/ml TNF-⍺ and 156 pg/ml (HIF-1⍺). All assays were performed according to the manufacturer’s protocols. All the samples were tested in duplicate. The quantity of cytokines was calculated based on a standard curve for each cytokine.
